# Identification and Correction of Sample Mix-Ups in Expression Genetic Data: A Case Study

**DOI:** 10.1534/g3.115.019778

**Published:** 2015-08-19

**Authors:** Karl W. Broman, Mark P. Keller, Aimee Teo Broman, Christina Kendziorski, Brian S. Yandell, Śaunak Sen, Alan D. Attie

**Affiliations:** * Department of Biostatistics and Medical Informatics, University of Wisconsin, Madison, Wisconsin 53706; † Department of Biochemistry, University of Wisconsin, Madison, Wisconsin 53706; ‡ Department of Statistics, University of Wisconsin, Madison, Wisconsin 53706; § Department of Horticulture, University of Wisconsin, Madison, Wisconsin 53706; ** Department of Epidemiology and Biostatistics, University of California, San Francisco, California 94107

**Keywords:** quality control, microarrays, genetical genomics, mislabeling errors, eQTL

## Abstract

In a mouse intercross with more than 500 animals and genome-wide gene expression data on six tissues, we identified a high proportion (18%) of sample mix-ups in the genotype data. Local expression quantitative trait loci (eQTL; genetic loci influencing gene expression) with extremely large effect were used to form a classifier to predict an individual’s eQTL genotype based on expression data alone. By considering multiple eQTL and their related transcripts, we identified numerous individuals whose predicted eQTL genotypes (based on their expression data) did not match their observed genotypes, and then went on to identify other individuals whose genotypes did match the predicted eQTL genotypes. The concordance of predictions across six tissues indicated that the problem was due to mix-ups in the genotypes (although we further identified a small number of sample mix-ups in each of the six panels of gene expression microarrays). Consideration of the plate positions of the DNA samples indicated a number of off-by-one and off-by-two errors, likely the result of pipetting errors. Such sample mix-ups can be a problem in any genetic study, but eQTL data allow us to identify, and even correct, such problems. Our methods have been implemented in an R package, R/lineup.

To map the genetic loci influencing a complex phenotype, one seeks to establish an association between genotype and phenotype. In such an effort, the maintenance of the concordance between genotyped and phenotyped samples and data is critical. Sample mislabeling and other sample mix-ups will weaken associations, resulting in reduced power and biased estimates of locus effects. In traditional genetic studies, one has limited ability to detect sample mix-ups and almost no ability to correct such problems. Inconsistencies between subjects’ sex and X chromosome genotypes may reveal some problems, and in family studies, some errors may be revealed through Mendelian inconsistencies at markers, but we will generally be blind to most errors.

In expression genetics studies, in which genome-wide gene expression is assayed along with genotypes at genetic markers, the presence of expression quantitative trait loci (eQTL) with profound effect on gene expression (particularly local eQTL, in which a polymorphism near a gene affects the expression of that gene) provides an opportunity to not just identify but also correct sample mix-ups.

In a mouse intercross with more than 500 animals and genome-wide gene expression data on six tissues, we identified a high proportion (18%) of sample mix-ups in the genotype data. We further identified a small number of mix-ups among the expression arrays in each tissue.

A number of investigators have developed methods for identifying such sample mix-ups (Westra *et al.* 2011; Ekstrøm and Feenstra 2012; Lynch *et al.* 2012; Schadt *et al.* 2012), and a similar approach was applied by Baggerly and Coombes (2008, 2009) in their forensic bioinformatics analyses of the Duke debacle. We have developed a further approach that is simple but effective. We illustrate its use through a particularly dramatic example.

## Materials and Methods

### Mice and genotyping

C57BL/6J (abbreviated B6 or B) and BTBR *T*^+^ *tf*/J (abbreviated BTBR or R) mice were purchased from the Jackson Laboratory (Bar Harbor, ME) and bred at the University of Wisconsin–Madison. The *Lep^ob^* mutation was introgressed into all strains using heterozygous parents to generate homozygous *Lep*^*ob/ob*^ offspring. F_2_ mice, all *Lep*^*ob/ob*^, were the offspring of F_1_ parents derived from a cross between BTBR females and B6 males (Supporting Information, Figure S1). F_2_ mice and a small number of parental and F_1_ controls were genotyped with the 5K GeneChip (Affymetrix).

### Gene expression microarrays

Gene expression was assayed with custom two-color, ink-jet microarrays manufactured by Agilent Technologies (Palo Alto, CA). RNA preparations were performed at Rosetta Inpharmatics (Merck & Co.). Six tissues were considered: adipose, gastrocnemius muscle (abbreviated gastroc), hypothalamus (abbreviated hypo), pancreatic islets (abbreviated islet), kidney, and liver. Tissue-specific messenger RNA (mRNA) pools were used for the second channel, and gene expression was quantified as the ratio of the mean log_10_ intensity (mlratio). For further details, see Keller *et al.* (2008).

### Sample mix-ups in the gene expression arrays

**Figure 1:**
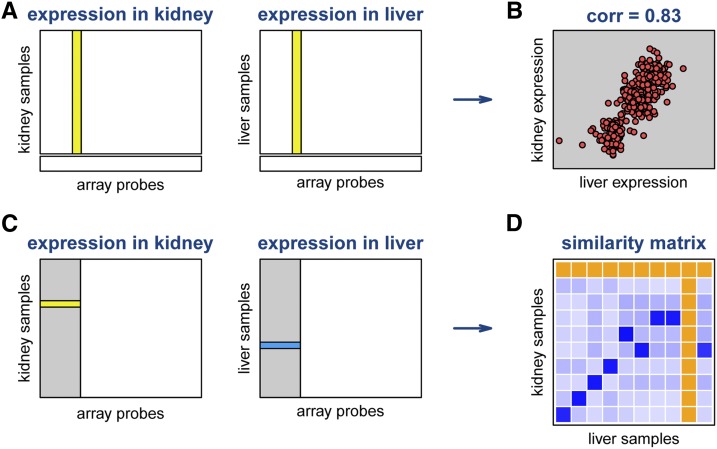
Scheme for evaluating the similarity between expression arrays for different tissues. We first consider the expression of each array probe for samples assayed for both tissues (A) and calculate the between-tissue correlation in expression (B). We identify the subset of array probes with correlation >0.75 (shaded region in C) and calculate the correlation in gene expression for one sample in the first tissue and another sample in the second tissue, across these selected probes. This forms a similarity matrix (D), for which darker squares indicate greater similarity. Orange squares indicate missing values (samples assayed in one tissue but not the other).

Let $x_{ip}^{s}$ denote the gene expression measure for sample *i* at array probe *p* in tissue *s*. We first considered each probe and each pair of tissues and calculated the between-tissue correlation across samples, omitting any samples with missing data for that probe in either tissue. We identified the subset of probes, for each tissue pair, with correlation >0.75. With this subset of probes, we then calculated the correlation between sample *i* in tissue *s* and sample *j* in tissue *t*; call it $r_{ij}^{st}$. As an illustration, consider the schematic in Figure 1: for each pair of tissues, we identified the subset of probes with high between-tissue correlation (the shaded region) and then evaluated the correlation between a sample in one tissue and another sample in the other tissue, across that subset of probes.

We then summarized the similarity between sample *i* in tissue *s* and sample *j* in the other tissues by the median correlation across tissue pairs that include tissue *s*, $r_{ij}^{s}=\text{median}\left\{r_{ij}^{st}:t\neq s\right\}$. Of course, we considered only pairs of tissues (*s*,*t*) for which sample *i* was measured in tissue *s* and sample *j* was measured in tissue *t*.

Sample mix-ups in tissue *s* were identified as samples *i* for which the self similarity, $r_{ii}^{s}$, was small, but for which there existed some array with high similarity: ${\text{max}}_{j\neq i}r_{ij}^{s}$ is large. We then inferred the correct label for sample *i* in tissue *s* to be $\arg\max_{j\neq i}r_{ij}^{s}$. In other words, viewing $r_{ij}^{s}$ as a similarity matrix, we were looking for rows with a small value on the diagonal, but with some large off-diagonal element in that row. To ensure confidence in the relabeling of such samples, we compared the maximum value in the row to the second-highest value.

To further investigate possible sample duplicates within a tissue, we considered the subset of probes with correlation >0.75 with at least one other tissue and then calculated between-sample correlations, across the chosen subset of probes, within that tissue.

### Sample mix-ups in the DNA samples

In our investigation of potential sample mix-ups in the DNA samples, we first calculated multipoint genotype probabilities at all markers and at pseudomarker positions between markers. The pseudomarker positions were placed at evenly spaced locations between markers, with a maximum spacing of 0.5 cM between adjacent markers or pseudomarkers. The multipoint genotype probability calculations were performed via a hidden Markov model, with an assumed genotyping error rate of 0.2% and with the Carter-Falconer map function (Carter and Falconer 1951).

We first considered each tissue, individually, and identified the subset of probes with a strong local eQTL. We considered all array probes with known genomic location and on an autosome, identified the nearest marker or pseudomarker to the location of the probe, and calculated a LOD score (log_10_ likelihood ratio) assessing the association between genotype at that location and the gene expression of that probe. The LOD score was calculated by Haley–Knott regression (Haley and Knott 1992), a quick approximation to standard interval mapping (Lander and Botstein 1989). Calculations were performed at a single location for each array probe, rather than with a scan of the genome. We chose the subset of probes with LOD >100.

Continuing to focus on one tissue at a time, we considered the set of local eQTL locations and the corresponding probe or probes. (Generally there was a single probe corresponding to a given eQTL location, but in a small number of instances for each tissue, there were a pair of probes at the same eQTL location; for islet, there were three eQTL with three corresponding probes, and for adipose there was one such trio.) For each eQTL position and for each mouse, we took the genotypes with maximal multipoint probability to be the observed eQTL genotype, provided that this exceeded 0.99; if no genotype had probability >0.99, the observed eQTL genotype was treated as missing.

Considering each eQTL in a tissue individually, we then formed a *k*-nearest neighbor classifier, with *k* = 40, for predicting eQTL genotype from the expression values for the corresponding probe or probes. For a given mouse, if more than 80% of the 40 nearest neighbors, by Euclidean distance, shared the same observed eQTL genotype, this was taken to be the inferred eQTL genotype for that mouse. If no more than 80% of the 40 nearest neighbors shared a common genotype, the inferred eQTL genotype was treated as missing.

To filter out samples that were clearly incorrect and improve our classifiers, we then calculated the proportion of matches, for each sample, between the observed eQTL genotypes and the corresponding inferred eQTL genotypes, omitted samples for which the proportion of matches was <0.7, and rederived the *k*-nearest neighbor classifiers with the subset of samples deemed likely correct.

**Figure 2:**
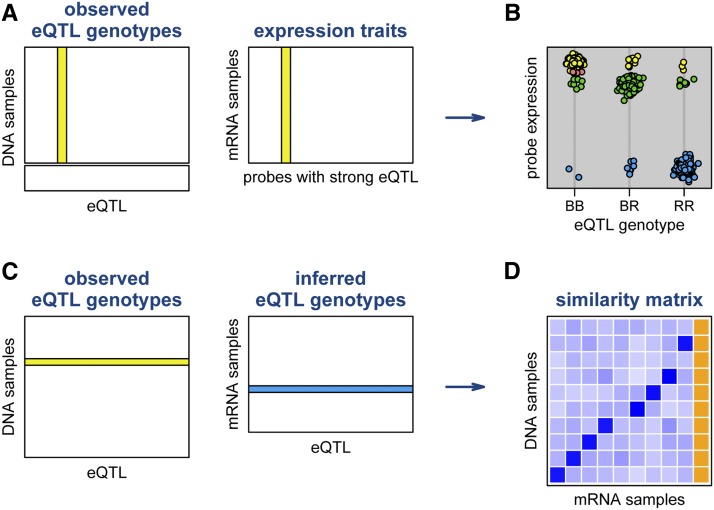
Scheme for evaluating the similarity between genotypes and expression arrays. We first identify a set of probes with strong local expression quantitative trait loci (eQTL). For each such eQTL, we use the samples with both genotype and expression data (A) to form a classifier for predicting eQTL genotype from the expression value (B). We then compare the observed eQTL genotypes for one sample to the inferred eQTL genotypes, from the classifiers, for another sample (C). The proportion of matches, between the observed and inferred genotypes, forms a similarity matrix (D), for which darker squares indicate greater similarity. Orange squares indicate missing values (for example, samples with genotype data but no expression data).

As an illustration, consider the schematic in Figure 2: for each tissue, we identified a subset of array probes with strong local eQTL, we derived classifiers for predicting eQTL genotype from the corresponding expression phenotypes, and then constructed a matrix of inferred eQTL genotypes. As a measure of similarity between a DNA sample and an mRNA sample, we calculated the proportion of matches between the observed eQTL genotypes for the DNA sample and the inferred eQTL genotypes for the mRNA sample.

To combine the tissue-specific similarity measures across the six tissues, we simply took the overall proportion of matching genotypes across all eQTL and across all tissues.

As in the investigation of sample mix-ups within the expression arrays, we treated the proportions of matches between observed and inferred eQTL genotypes as a similarity matrix. Problem DNA samples were identified as rows for which the value on the diagonal (the self similarity) was small. In such rows, we inferred the correct label to be that of the maximal off-diagonal value, provided that this maximum was large and was well above the second-largest value.

### QTL analysis

To characterize the improvement in results after correction of sample mix-ups, we performed QTL analysis with several traits of interest, including the expression traits in each tissue, with the original data and with the corrected data. In the corrected data, we omitted the DNA samples that could not be verified to be correct (that is, those with no corresponding gene expression data.)

#### Insulin:

We first considered a clinical phenotype of considerable interest: 10-week plasma insulin. QTL analysis was performed by Haley−Knott regression (Haley and Knott 1992), with log insulin, and with sex included as an interactive covariate (that is, allowing the effects of QTL to be different in the two sexes).

#### Agouti and tufted coat:

We considered two simple Mendelian traits: agouti coat color (due to a single gene on chromosome 2) and tufted coat (due to a single gene on chromosome 17). QTL analysis was performed treating each phenotype as a binary trait (Xu and Atchley 1996; Broman 2003). To handle possible marker genotyping errors at the causal loci, we took the observed genotypes to be those with maximal multipoint probability, provided that this exceeded 0.99; if no genotype had probability >0.99, the observed genotype was treated as missing.

#### eQTL analyses:

We considered each of the six tissues individually, and focused on the subset of probes with known genomic location on an autosome or the X chromosome. For hypothalamus tissue, we omitted a batch of 119 poorly behaved arrays, although these had been included in our efforts to identify sample mix-ups.

Expression measures were transformed to normal quantiles. That is, the expression measures were converted to ranks $R_{i}\in \left\{1,\ldots ,n\right\}$ and then transformed to $y_{i}=\Phi^{-1}\left[\left(R_{i}-0.5\right)/n\right]$, where *Φ*^−1^ is the inverse of the normal cumulative distribution function.

QTL analysis was performed by Haley−Knott regressions with sex included as an interactive covariate. We considered the maximal peak for each array probe on each chromosome and inferred the presence of a QTL if the LOD score exceeded 5, a 5% genome-wide significance level established by computer simulation. An inferred eQTL was considered a local eQTL if the 2-LOD support interval contained the genomic location of the corresponding array probe; otherwise, it was considered a *trans*-eQTL.

### Software

All analyses were conducted with R (R Development Core Team 2015). QTL analyses were performed with the R package, R/qtl (Broman *et al.* 2003). Our methods for identifying sample mix-ups have been assembled as an R package, R/lineup, available at http://github.com/kbroman/lineup as well as The Comprehensive R Archive Network (CRAN; http://cran.r-project.org).

### Data availability

The genotype and gene expression microarray data are available at the QTL Archive, now part of the Mouse Phenome Database: http://phenome.jax.org/db/q?rtn=projects/projdet&reqprojid=532.

## Results

We first became aware of potential problems in the samples through the identification of six duplicate DNA samples and 32 mice whose X chromosome genotypes were incompatible with their sex. We genotyped 554 F_2_ mice at 2060 informative SNPs, including 20 on the X chromosome. Three samples were assigned “no call” at all markers and not considered further. Six pairs were seen to be duplicates, with over 98% genotype identity across typed markers (Table S1).

The F_2_ mice were the offspring of F_1_ siblings derived by crossing BTBR females to B6 males (Figure S1). F_2_ females should be homozygous BTBR (RR) or heterozygous (BR) on the X chromosome; F_2_ males should be hemizygous B or R. (Note that homozygous and hemizygous genotypes could not be distinguished.) However, 19 females exhibited some homozygous B6 genotypes on the X, and 17 males exhibited some heterozygous genotypes (Figure S2). Although four of these males had a single heterozygous genotype that was likely a genotyping error, the 19 females and the other 13 males were clearly indicated to have swapped sex. There were an additional 53 females and 50 males with homozygous RR or hemizygous R genotypes for all markers on the X chromosome, compatible with either sex.

In cleaning the genotype data, we omitted a set of seven samples, including one pair of the sample duplicates, with poorly behaved data. (They showed a high rate of apparent genotyping errors, an unusually large proportion of homozygous genotypes, and an unusually large number of apparent crossovers.) For the other five pairs of duplicates, we omitted one sample from each pair.

### Sample mix-ups in the gene expression arrays

For each of six tissues (adipose, gastroc, hypo, islet, kidney, liver), approximately 500 F_2_ mice were assayed for gene expression with two-color Agilent arrays with tissue-specific pools (Table S2). A small number of poorly behaved arrays were omitted. We later discovered a batch of 119 poorly behaved arrays for hypo, but these were included in the analyses described here. There were 527 mice assayed for at least one of the six tissues, but not all mice were assayed for all tissues. In particular, there were 27 mice assayed only for gene expression in kidney, and 43 mice assayed for all tissues except kidney. Furthermore, 27 mice were genotyped but were not subject to gene expression analysis.

To identify potential sample mix-ups among gene expression arrays, we first identified, for each pair of tissues, a subset of array probes with high between-tissue correlations. Consideration of all probes would greatly reduce the apparent correlation between arrays, due to the abundance of unexpressed genes. For example, for Mouse3567, the correlation between gene expression in kidney and in liver, across all 40,572 probes, is 0.32, whereas for the subset of 155 probes with correlation >0.75 between kidney and liver, the correlation is 0.78. (See Figure S3.)

Figure S4 contains density estimates of the between-tissue correlations for all array probes. The densities are organized by tissue, with the panel for each tissue containing the five tissue pairs involving that tissue. There are some small differences among tissue pairs, but the vast majority of between-tissue correlations are between −0.25 and 0.50. Table S3 contains the numbers of probes for each pair of tissues with correlations exceeding 0.70, 0.75, 0.80, and 0.90, respectively. We focused on probes with correlations >0.75, of which there were between 46 and 200 probes per tissue pair.

For each pair of tissues, we calculated the correlations among samples across the subset of correlated probes. For each tissue, we then summarized the similarity between each sample in that tissue and each sample in other tissues by the median correlations, across the tissue pairs that included the target tissue.

Figure S5 contains histograms of the similarity measures for each tissue, separating the self-self similarities (the diagonal elements) and the self-nonself similarities (the off-diagonal elements). There are a number of clear outliers: small self-self similarities and large self-nonself similarities. The self-nonself similarities follow a bimodal distribution, with the lower mode corresponding to opposite-sex pairs and the upper mode corresponding to same-sex pairs. The chosen probes included a probe in *Xist* (involved in X chromosome inactivation) and probes on the Y chromosome.

**Figure 3:**
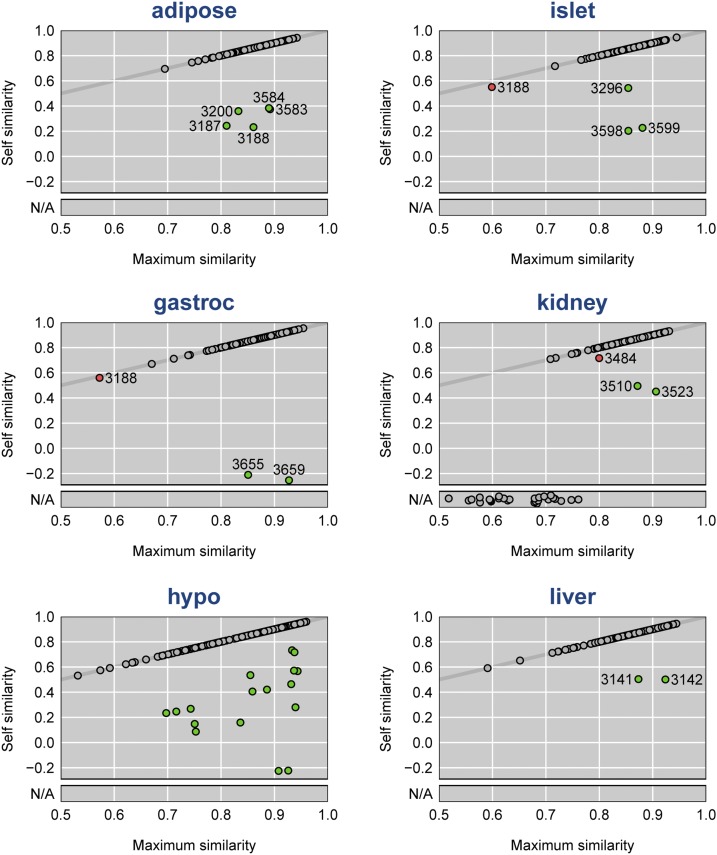
Self similarity (median correlation across tissue pairs) *vs.* maximum similarity for the expression arrays for each tissue. The diagonal gray line corresponds to equality. Green points are inferred to be sample mix-ups. Gray points correspond to arrays for which the self similarity is maximal. Red points correspond to special cases (see the text). There were 27 samples assayed only for kidney; these have missing self similarity values.

To identify problem samples in each tissue, we considered for each sample, the self similarity *vs.* the maximum similarity (that is, the values on the diagonal of the similarity matrix and the maximum values in each row). These are displayed in Figure 3.

The vast majority of samples in each tissue were indicated to be correctly labeled: the self similarity was the maximum similarity. But for each tissue, there were at least a few samples that were more like some other sample in the other tissues. In each case, we infer the correct label to be that with the maximal similarity. In Figure S6, we display the second-highest similarity *vs.* the maximum similarity for each sample in each tissue. The problem samples (colored green) are generally well away from the diagonal, indicating good support for our ability to infer the correct label.

The red dots in Figure 3 and Figure S6 are special cases: The Mouse3188 sample is highlighted as a potential problem in both islet and gastroc (being slightly off the diagonal line), but this is because that sample was involved in array swaps in two different tissues (adipose and hypo). This is the only sample indicated to be mislabeled in multiple tissues. We also highlight Mouse3484 in gastroc, which appeared to be a mixture (more in the paragraphs to follow).

**Figure 4:**
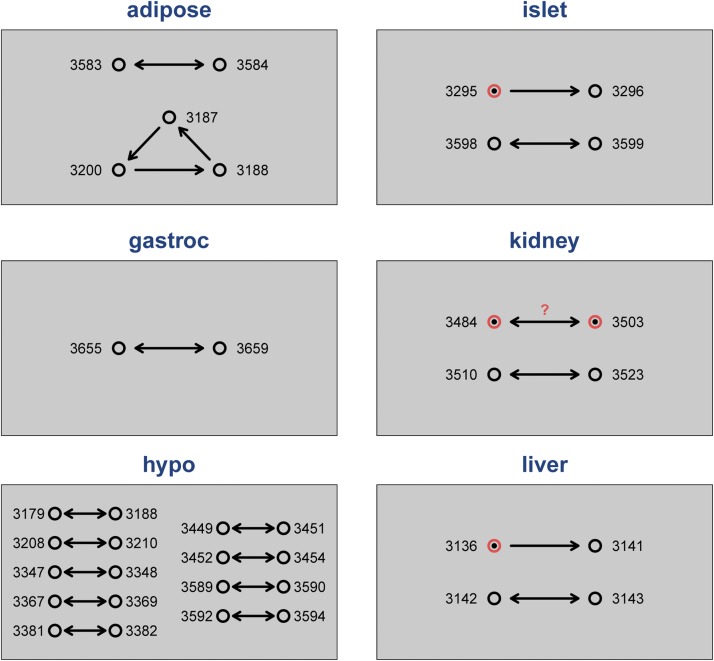
The messenger RNA sample mix-ups for the six tissues. Double-headed arrows indicate a sample swap. The trio of points in adipose corresponds to a three-way swap. The pink circles with a single-headed arrow, in islet and liver, are sample duplicates. The questionable case in kidney indicates a potential sample mixture arrayed twice.

The inferred errors are displayed in Figure 4. For adipose, we identified two problems. The samples for Mouse3583 and Mouse3584 were swapped, and there was a three-way swap among Mouse3187, Mouse3188, and Mouse3200, with the sample labeled Mouse3187 really being Mouse3188, that labeled Mouse3188 really being Mouse3200, and that labeled Mouse3200 really being Mouse3187.

For gastroc, there was a single sample swap, between Mouse3655 and Mouse3659. For hypo, there were nine pairs of sample swaps. For islet, the samples Mouse3598 and Mouse3599 were swapped, and the sample labeled Mouse3296 was really a duplicate (or *unintended technical replicate*) of the Mouse3295 sample. For liver, the sample labeled Mouse3142 really corresponded to Mouse3143 (Mouse3142 was not assayed for gene expression in liver), and the sample labeled Mouse3141 was really a duplicate of the Mouse3136 sample.

For kidney, the samples for Mouse3510 and Mouse3523 were swapped, and Mouse3484 also was seen to be a problem. We believe that the samples for Mouse3484 and Mouse3503 may have been mixed and assayed twice in duplicate (more in the paragraphs to follow). There were 27 samples that were assayed for gene expression only in kidney; for these, the self similarity cannot be calculated. We have limited ability to detect mix-ups for these samples, but none were very close to any sample in other tissues, and so they can, at least provisionally, be assumed to be correctly labeled.

To further illustrate the sample swaps, Figure S7 contains scatter plots of the gastroc arrays labeled Mouse3655 and Mouse3659 against the arrays in the other tissues with those labels. For each pair of tissues, we plot the array probes with between-tissue correlation >0.75. Mouse3655 in gastroc is correlated with Mouse3659 in other tissues, whereas Mouse3659 in gastroc is correlated with Mouse3655 in other tissues, indicating a clear swap between these samples within gastroc.

Figure S8 contains similar scatter plots for a pair of inferred duplicates, with the sample labeled Mouse3141 in liver really being a duplicate of the Mouse3136 liver sample. Mouse3136 liver and Mouse3141 liver are each correlated with Mouse3136 in other tissues and not with Mouse3141, and the two samples are extremely highly correlated with each other (see the two central panels in the bottom row). In Figure S9, we display the between-sample correlations for samples with these two labels, for all pairs of tissues, with the pairs including liver highlighted in red. The Mouse3136 samples are correlated for all tissue pairs; the Mouse3141 samples are correlated for all tissue pairs not involving liver, and the Mouse3141 liver sample is correlated with all Mouse3136 samples in other tissues.

The Mouse3484 and Mouse3503 samples in kidney appear to be sample duplicates, but these samples are correlated with each of Mouse3484 and Mouse3503 in the other tissues. We’re inclined to believe that the two kidney samples were mixed and arrayed in duplicate, but we are not able to prove this point. Figure S10 contains scatter plots for the two samples in kidney *vs.* all tissues; the central panels in the second row from the bottom indicate that the two samples are highly correlated and so likely replicates, but all scatter plots here show strong correlation. Figure S11 contains the between-sample correlations for both sample labels in all tissue pairs; contrast this with Figure S9, for the simple duplicate in liver. Mouse3484 kidney and Mouse3503 kidney are strongly correlated with both samples in the other tissues but not so strongly as Mouse3484 and Mouse3503 are with themselves in the nonkidney pairs. And for tissue pairs not including kidney, Mouse3484 and Mouse3503 are much more weakly correlated.

Because we were unable to resolve the problems with Mouse3484 and Mouse3503 in kidney, these two arrays were omitted from later analyses. The two simple sample duplicates, one in islet and one in liver, were combined and assigned the correct label. The other sample mix-ups were relabeled as inferred in Figure 4.

Expression of the *Xist* gene (involved in X chromosome inactivation and so highly expressed in females but not males) and of genes on the Y chromosome is a useful diagnostic for the sex of an mRNA sample. In Figure S12, we display, for each tissue, the average expression across four Y chromosome genes *vs.* the expression of *Xist*, with the original data and after correction of the sample mix-ups in the expression arrays. Just three of the sample-swaps (one in gastroc and two in hypo) involved opposite-sex pairs. These show up clearly in the left column, with the original data, and are resolved after correction of the sample mix-ups. The unusual pattern of expression in hypo, with a bimodal distribution for the Y chromosome genes in males and a large number of females with relatively low *Xist* expression, was due to a set of 119 poorly behaved arrays.

### Sample mix-ups in the genotypes

Having corrected the sample mix-ups among the gene expression arrays, we turned to potential problems in the genotypes. For each tissue, we considered the 36,364 autosomal array probes with known genomic location and identified those with a strong local eQTL, having LOD score >100 for the association between the probe expression measures and genotype at the corresponding location.

**Figure 5:**
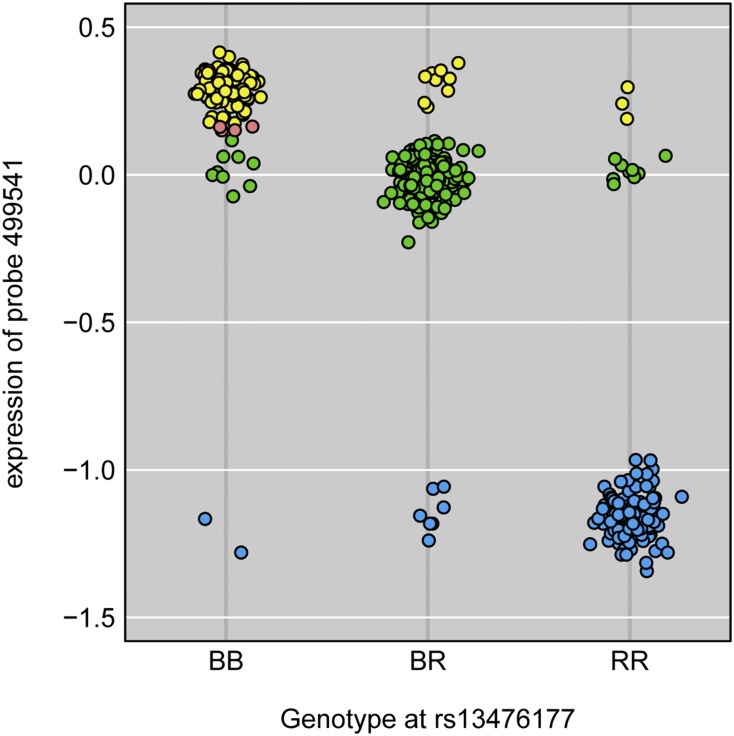
Plot of islet expression *vs.* observed genotype for an example probe. Points are colored by the inferred genotype, based on a k-nearest neighbor classifier, with yellow, green, and blue corresponding to BB, BR, and RR, respectively, where B = B6 and R = BTBR. Salmon-colored points lie at the boundary between two clusters and were not assigned.

For each such probe, we created a *k*-nearest neighbor classifier (with k = 40), for predicting eQTL genotype from the expression phenotype. For example, in Figure 5, we display the expression, in islet, of probe 499541 (on chromosome 1) *vs.* genotype at the nearest marker. At this probe, there are three clear groups of mice, with B6 homozygotes (BB) having high expression, BTBR homozygotes (RR) having low expression, and heterozygotes (BR) intermediate. There are a number of mice whose expression does not match their observed eQTL genotype; the classifier infers a different eQTL genotype. The points highlighted in pink have expression at the boundary between the BB and BR groups and are left unassigned. (To assign an inferred eQTL genotype to a point, we required that 80% of the nearest neighbors had a common eQTL genotype.)

For sets of probes mapping to approximately the same genomic location, we considered the probes’ expression jointly. Examples of pairs of probes mapping to the same location are shown in Figure S13, with points colored by observed eQTL genotype.

We considered 45–115 eQTL per tissue; their locations on the genetic map of markers is shown in Figure S14. The majority of eQTL had a single corresponding probe. There were 3–14 eQTL per tissue with a pair of corresponding probes. For islet, there were three eQTL with three corresponding probes, and for adipose there was one such trio.

For each tissue, we calculated the proportion of matches between the observed eQTL genotypes for each DNA sample and the inferred eQTL genotypes from each mRNA sample, as a measure of similarity between the DNA and mRNA samples. We further calculated a combined measure of similarity as the overall proportion of matches, pooling all six tissues.

Figure S15 contains histograms of the similarity measures for each tissue, separating the self-self similarities (the diagonal elements) and the self-nonself similarities (the off-diagonal elements). There are a number of clear outliers: small self-self similarities and large self-nonself similarities.

**Figure 6:**
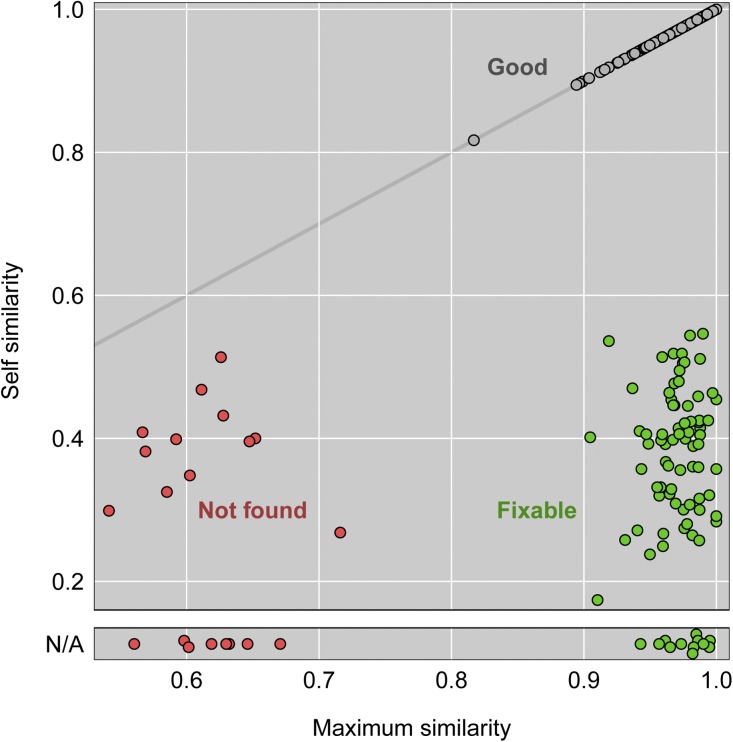
Self-similarity (proportion matches between observed and inferred expression quantitative trait loci genotypes, combined across tissues) *vs.* maximum similarity for the DNA samples. The diagonal gray line corresponds to equality. Samples with missing self similarity (at bottom) were not intended to have expression assays performed. Gray points correspond to DNA samples that were correctly labeled. Green points correspond to sample mix-ups that are fixable (the correct label can be determined). Red points comprise both samples mix-ups that cannot be corrected as well as samples that may be correct but cannot be verified as no expression data are available.

To identify problem DNA samples, we again considered the self similarity *vs.* the maximum similarity (that is, the values on the diagonal of the similarity matrix *vs.* the maximum values in each row). Figure 6 contains a scatterplot of these values. Gray points, with maximum similarity equal to the self similarity, are inferred to be corrected labeled. Green points, with small self similarity but large maximum similarity, are inferred to be incorrect, but are fixable. Red points concern DNA samples for which no corresponding mRNA sample can be found.

Detailed results for the six tissues, with tissue-specific similarity values, are shown in Figure S16. The points are colored as in Figure 6, based on the combined similarity measure. The points with missing self similarity (at the bottom of each panel) were not intended to be assayed for gene expression in that tissue. The tissue-specific results are concordant with the overall conclusions, with two caveats. First, there are a number of green points (corresponding to mislabeled, but fixable, DNA samples), with low maximum similarity in each tissue. These correspond to samples for which gene expression assays were not performed for that tissue, the bulk of which are for the 27 samples that were assayed only for gene expression in kidney and the 43 samples that were assayed for all tissues except kidney. Second, for hypo, the strength of eQTL associations were weaker, and fewer eQTL were considered, than for the other tissues, and so there is less separation between the green and pink points.

In Figure S17, we display the second-highest similarity *vs.* the maximum similarity, for the combined similarity measures accounting for all tissues. The fixable mislabeled samples (in green) are all well away from the diagonal, indicating good support for our ability to infer the correct label.

**Figure 7:**
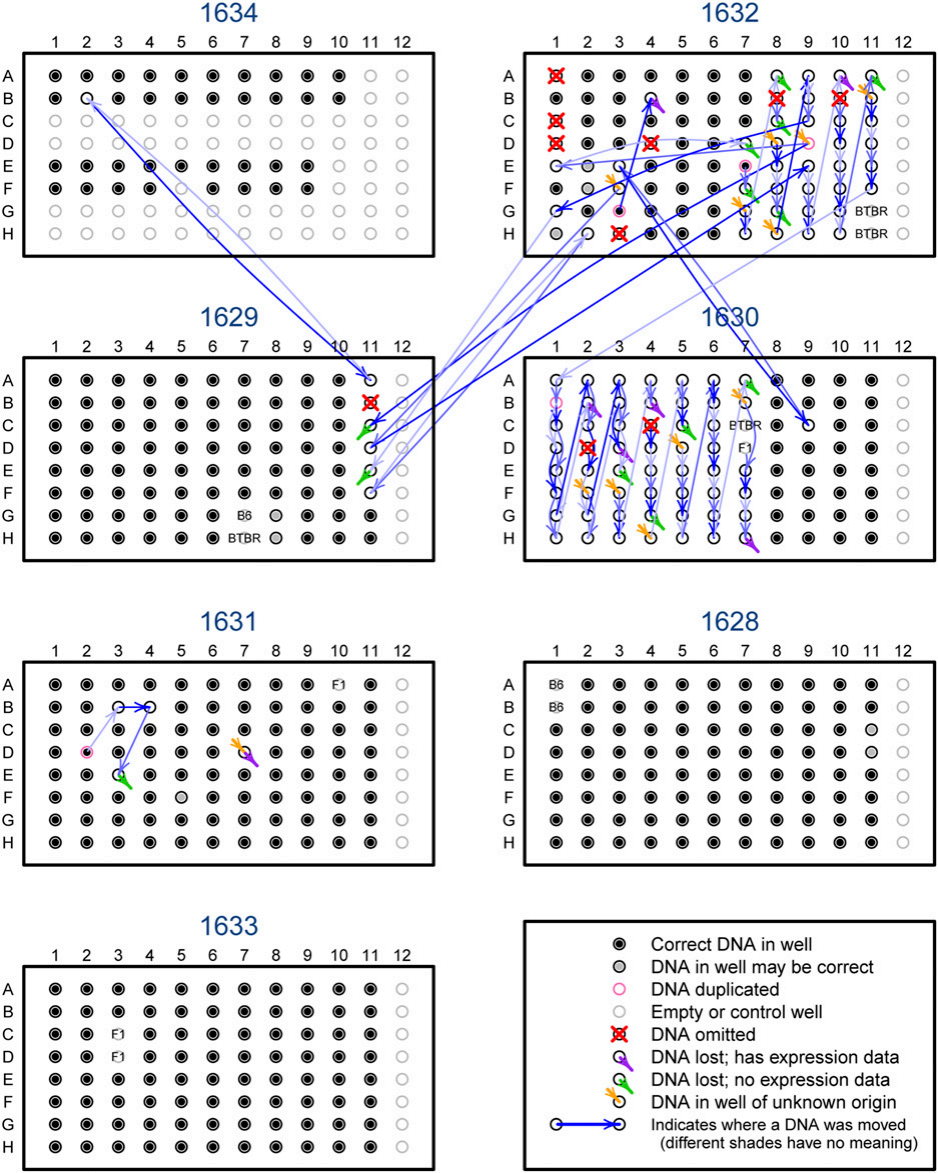
The DNA sample mix-ups on the seven 96-well plates used for genotyping. Black dots indicate that the correct DNA was put in the well. Blue arrows point from where a sample should have been placed to where it was actually placed; the different shades of blue convey no meaning. Red X’s indicate DNA samples that were omitted. Orange arrowheads indicate wells with incorrect samples, but the sample placed there is of unknown origin. Purple and green arrowheads indicate cases in which the sample placed in the well was incorrect, but the DNA that was supposed to be there was not found; with the purple cases, there was corresponding gene expression data, while for the green cases, there was no corresponding gene expression data. Pink circles (*e.g.*, well D02 on plate 1631) indicate sample duplicates. Gray dots indicate that the sample placed in the well cannot be verified, as there was no corresponding gene expression data. Gray circles indicate controls or unused wells.

The inferred mix-ups among the DNA samples are displayed in Figure 7 according to the arrangement of the samples on the 96-well genotyping plates. Black dots indicate that the correct DNA sample was placed in the correct well. The blue arrows point from the well in which a DNA sample was supposed to be placed, to the well where it was actually placed. For example, on plate 1631, the sample in well D02 was placed in the correct well but was also placed in well B03. The sample that belonged in B03 was placed in B04, the sample that belonged in B04 was placed in E03, and the sample belonging in E03 was not found (but, as indicated by the green arrowhead, there was no corresponding gene expression data).

Although there were many long-range sample swaps, particularly for samples belonging in the eleventh column of plate 1629, the bulk of the errors occurred on plates 1632 and 1630, with a long series of off-by-one and off-by-two errors indicative of single-channel pipetting mistakes.

Let us describe a small portion of the further errors. On plate 1632, the sample belonging in well E07 was placed in the correct well but was also placed in the well below, F07. The sample belonging in well F07 was not found but had no corresponding gene expression data. The sample placed in well G07 was incorrect but had no corresponding gene expression data, and so presumably corresponds to that which should have been in the well above, F07. The sample belonging in well G07 was placed one below, H07. There are then a series of off-by-one errors, except that the sample belonging in well C09 was actually placed in well G01, while the sample belonging in well D09 was placed in both well E01 and on plate 1629 (well C11).

Of the 554 DNA samples that were genotyped, 10 were omitted due to poorly behaved genotypes (including a pair of replicates), 435 were found to be correctly labeled, and 8 were possibly correct but could not be verified due to lack of gene expression assays. However, five samples were duplicates of other samples, 84 were incorrectly labeled but the correct label could be assigned, and 12 were incorrectly labeled and the correct label could not be identified. Thus, at least 18% of the samples were involved in sample mix-ups.

We had initially become suspicious of possible sample mix-ups through the identification of 36 mice whose X chromosome genotypes were inconsistent with their sex. After correction of the sample mix-ups, there were no such discrepancies. Only a small portion of the problems were identified through such sex/genotype incompatibilities, because the majority of sample mix-ups were off-by-one errors in the genotype plates, and the samples were arranged on the plates so that adjacent samples were often the same sex.

The large discrepancies between expression and eQTL genotype seen in Figure 5 and Figure S13 are largely eliminated after correction of the inferred sample mix-ups. Figure S18 shows the same examples but with the corrected data. Figure S18, A−D correspond to the panels in Figure S13; the genotypes are now more clearly separated, although some overlap remains and there are a few outliers (most notably, in Figure S18B). Figure S18E corresponds to Figure 5; after correction of the sample mix-ups, there is no overlap between the three genotype groups.

### QTL mapping results

**Figure 8:**
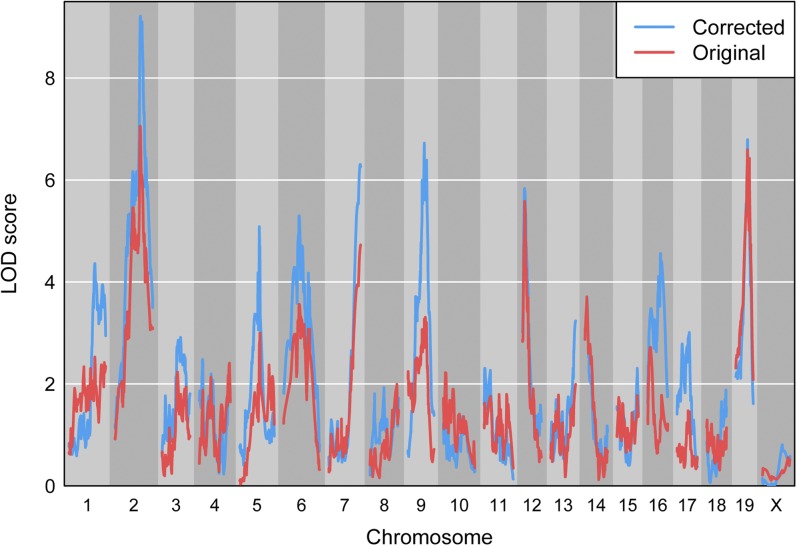
LOD curves for 10-week insulin level, before (red) and after (blue) correction of the sample mix-ups.

It should come as no surprise that the correction of the sample mix-ups, particularly the 18% mix-ups in the DNA samples, leads to great improvement in QTL mapping results. Figure 8 contains LOD curves for 10-week insulin level with the original and corrected datasets. With the original data, four chromosomes had LOD score >4; after correction of the sample mix-ups, nine chromosomes have LOD score >4.

Two coat-related traits were recorded for the F_2_ mice: agouti and tufted coats. Concerning the agouti coat: BTBR mice have tan coats, whereas B6 mice are black; this is due to a gene on chromosome 2, and the BTBR allele is dominant. Mapping the agouti coat color as a binary phenotype, the LOD score on chromosome 2 increased from 64 to 110 after correction of the sample mix-ups (Figure S19A). Although the corrected data still contained inconsistencies between genotype and coat color, the number of inconsistencies decreased from 47 to 7 (Table S4).

Tufted coat is due to a single gene on chromosome 17, with the BTBR allele (with the tufted phenotype) being recessive to the B6 allele (not tufted). Mapping this phenotype as a binary trait, the LOD score on chromosome 17 increased from 64 to 107 after correction of the sample mix-ups (Figure S19B). Although, as with agouti, the corrected data still contained inconsistencies between genotype and phenotype, the number of inconsistencies decreased from 37 to 4 (Table S5).

**Figure 9:**
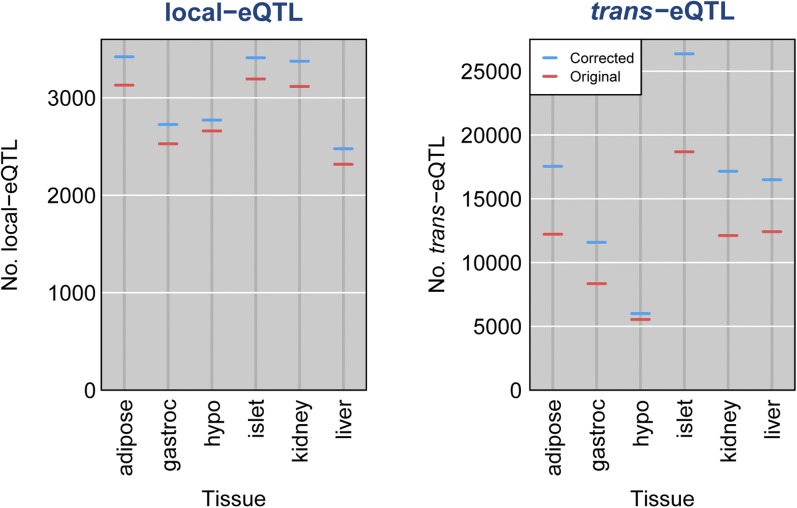
Numbers of identified local and *trans*-eQTL with LOD ≥ 5, with the original data (red) and after correction of the sample mix-ups (blue), across 37,797 array probes with known genomic location. An expression quantitative trait loci (eQTL) was considered local if the 2-LOD support interval contained the corresponding probe; otherwise it was considered *trans*.

Finally the corrected data resulted in a great increase in the numbers of inferred eQTL in the six tissues (Figure 9). For each array probe with known genomic position, we performed a genome scan, including sex as an interactive covariate (that is, allowing the QTL effect to be different in the two sexes). For each array probe, we counted the number of chromosomes with a peak LOD score above 5. Such a peak, on the chromosome containing the probe, was considered a local eQTL if the 2-LOD support interval contained the probe location; other peaks were called *trans*-eQTL. The inferred number of local eQTL increased by 7% across tissues (with a somewhat-smaller increase in hypo). The inferred number of *trans*-eQTL increased by 37% across tissues (although only by 8% in hypo). The modest increases in hypo were due in part to the omission of 119 poorly behaved arrays. The increased numbers of inferred eQTL is also seen with more stringent thresholds; the numbers of eQTL with LOD ≥ 10 are shown in Figure S20.

## Discussion

In a mouse intercross with more than 500 animals and gene expression microarray data on six tissues, we identified and corrected sample mix-ups involving 18% of the DNAs, along with a small number of mix-ups in each batch of expression arrays. The QTL mapping results improved markedly after the correction of mix-ups, but it was perhaps most surprising just how strong the results were before the corrections.

To align the expression arrays, we first identified subsets of genes with strong between-tissue correlation in expression and then considered the correlations between samples across these subsets of genes. To align genotypes and expression arrays, we identified transcripts with strong local eQTL, formed predictors of eQTL genotype from expression values, and calculated the proportion of matches between the observed eQTL genotypes for a DNA sample and the predicted eQTL genotypes for an mRNA sample.

This approach applies quite generally: Whenever one has two data matrices, *X* and *Y*, whose rows should correspond, one should check that the rows do in fact correspond. The simplest approach is to first identify subsets of associated columns (in which a column of *X* is associated with a column of *Y*) and then calculate some measure of similarity between rows of *X* and rows of *Y*, across that subset of columns.

Similar approaches have been described by a number of groups. Westra *et al.* (2011) considered a number of public datasets and found an overall rate of 3% sample mix-ups, with one dataset (Choy *et al.* 2008) having 23% mix-ups. Schadt *et al.* (2012) showed that, with the tight connection between genotypes and gene expression phenotypes, external eQTL information can, in principle, be used to identify individuals participating in a gene expression study: Genome-wide gene expression is just as revealing of individual identities as genome-wide genotype data. Lynch *et al.* (2012) highlighted issues arising in large tumor studies and focused particularly on a number of experimental design issues, such as plate layout. Ekstrøm and Feenstra (2012) considered the identification of sample mix-ups in genome-wide association studies, focusing on a small number of phenotypes, such as blood group data, with strong genotype-phenotype associations. Also relevant is the forensic bioinformatics work of Baggerly and Coombes (2008, 2009), particularly their efforts to correct mix-ups in data files. Finally, Jun *et al.* (2012) recently described methods for detecting mixtures in DNA samples based on genotype or sequencing data, and there is considerable work on detecting mislabeled microarrays (*e.g.*, Zhang *et al.* 2009; Bootkrajang and Kabán 2013).

There are a number of opportunities for improvement in our approach. In particular, a number of critical parameters (such as the LOD score for choosing eQTL, and the number of nearest neighbors and the minimum vote in the *k*-nearest neighbor classifier) were chosen in an *ad hoc* way. The choice of such parameters influences the variation within and the separation between the self-self and self-nonself distributions of similarity measures, and thus our ability to identify errors. In addition, other classification methods might be used, although the *k*-nearest neighbor classifier has an important advantage: It works well even in the presence of misclassification error in the “training” data.

Perhaps the most important lesson from this work is the value of investigating aberrations. One should follow up any observed inconsistencies in data, to identify the source. In particular, one should not rely solely on LOD scores or other summary statistics, but also inspect plots of genotype *vs.* phenotype, such as that in Figure 5.

Of course, there are many possible errors that we couldn’t see by these approaches. For example, all of the tissues (including the DNA) for a pair of animals might be swapped, or there may be mix-ups within the clinical phenotypes (such as plasma insulin levels). And some mix-ups are detectable but not correctable.

We have not identified any between-tissue mix-ups in the expression data, but such errors are possible. For that type of error, it may be useful to consider the gene expression bar code developed by Zilliox and Irizarry (2007).

The correction of inferred sample mix-ups, as we have done, may introduce bias toward larger estimated eQTL effects. We believe that, in the current study, there is little risk of such bias, because the data provide strong evidence for specific sample labels. If the correction of sample mix-ups were accompanied by a greater level of uncertainty, one might consider omitting samples rather than assigning the inferred labels, though such an approach could also incur some bias.

Finally, one might ask, following these findings: What is an acceptable error rate in a research study? And what laboratory procedures should be instituted to avoid such errors? There exist procedures to help protect against errors, both for genotypes (*e.g.*, Huijsmans *et al.* 2007a,b) and for microarrays (Grant *et al.* 2003; Imbeaud and Auffray 2005; Walter *et al.* 2010), but they are not always put into practice. However, as the current study indicates, with expression genetic data, one can accommodate a high rate of errors provided that one applies appropriate procedures to detect and correct such errors.

## 
